# Impact of a Quality Improvement Initiative to Optimize the Discharge Process of Pediatric Gastroenterology Patients at an Academic Children’s Hospital

**DOI:** 10.1097/pq9.0000000000000213

**Published:** 2019-09-27

**Authors:** Joseph A. Moo-Young, Francisco A. Sylvester, Ria D. Dancel, Sheryl Galin, Heidi Troxler, Kathleen K. Bradford

**Affiliations:** From the * Department of Pediatrics, University of North Carolina School of Medicine, Chapel Hill, N.C.; † North Carolina Children’s Hospital, University of North Carolina Medical Center, Chapel Hill, N.C.

## Abstract

Discharge is an important and complex process that can be optimized to reduce inpatient healthcare inefficiency and waste. This study aimed to increase the percentage of patients discharged before 1 pm by 20% from an academic inpatient pediatric gastroenterology service (IPGS), over 6 months.

**Methods:**

We conducted a preintervention and postintervention study of patients discharged from IPGS. Patients discharged from January to June 2016, and those following our intervention from June to December 2016, were studied. Interventions included (1) implementation of the electronic medical record medical and logistical discharge criteria checklists for the 4 most common IPGS discharge diagnoses, (2) standardization of the rounds process to prioritize discharge, (3) education of nursing staff and families about the role they played in discharge. Process, outcome, and balancing measures were analyzed.

**Results:**

Three hundred fifty-five total discharges were studied. Between the preintervention and postintervention groups, there were no significant improvements in discharge order time, physical discharge time, discharge response time, or discharges before 1 pm. The balancing measure of 30-day readmission was unaffected. However, length of stay (LOS) index, calculated as the ratio of actual to expected LOS, improved; when translated into days, LOS declined by 1 day, with potential associated savings of $373,000.

**Conclusions:**

Interventions to improve discharge timeliness on IPGS service demonstrated mixed effectiveness. Only LOS index improved. Further iterative quality improvement interventions are needed to continue optimizing discharge timeliness and change the culture of pediatric discharge on inpatient subspecialty services in academic children’s hospitals.

## INTRODUCTION

In 2014, inpatient hospital care in the United States accounted for $971.8 billion out of $3.0 trillion in total healthcare spending,^1^ and hospitalized children represent a rapidly growing proportion of expenditures.^2^ As US healthcare costs continue rising,^3^ healthcare enterprises are focusing on eliminating inpatient operational inefficiencies and waste to reduce unnecessary costs.

Discharge is an important and complex inpatient process that can be optimized. Prior research has shown nearly 1 in 4 patients in a large academic pediatric hospital study population experienced a medically unnecessary prolongation in their length of stay (LOS) of at least 1 day, with a mean delay of 2.1 days. This result accounts for 8.9% of patient costs.^4^ Timely discharges are the focus of quality and cost reduction initiatives as earlier hospital discharges can improve hospital throughput, reduce bottlenecks, alleviate bed scarcity, and increase patient and staff satisfaction while decreasing LOS and cost.^5,6^

Previous work identified physician behavior and discharge planning as major contributors to medically unnecessary discharge delays.^7^ Interventions such as administrative changes^8^ and electronic medical record (EMR) discharge criteria checklists^4,9^ have been demonstrated to improve discharge timeliness and address delay factors.

Our institution identified the pediatric discharge process as a quality gap, in part, due to concerns about increased demand for inpatient beds in the setting of limited hospital capacity. Previous local quality improvement pilots successfully improved discharge timeliness for the general pediatric hospitalist service. However, no such work had been done for the inpatient pediatric gastroenterology service (IPGS). IPGS leadership was interested in improving the discharge process due to high patient volume, frustration with discharge delays, and recognition that improvements could be highly impactful on hospital throughput. IPGS had the second-highest acute care inpatient census, after the general pediatric hospitalist service. Furthermore, in published literature, previous pediatric discharge improvement studies focused on general inpatient services, but not subspecialty services.

## METHODS

### Study Setting

The setting for this preintervention and postintervention study was the academic IPGS of North Carolina Children’s Hospital at the University of North Carolina Medical Center. IPGS admits pediatric patients requiring acute gastroenterology inpatient care to this 150-bed tertiary care hospital. The University of North Carolina Institutional Review Board approved the study.

### Context

IPGS patients were hospitalized with a wide range of digestive system problems, from functional disorders to inflammatory bowel diseases. Some patients were chronically ill and required continued posthospital support from home health services and durable medical equipment suppliers. The IPGS multidisciplinary rounding team consisted of 1 attending physician, 1 pediatric nurse practitioner, the bedside nurse, 3 resident physicians, 2 medical students, and a nutritionist and pharmacist as needed.

Five pediatric gastroenterologists shared rounding duties, and each IPGS attending physician rounded with IPGS 1 week at a time. A pediatric nurse practitioner, known as a Ward Team Coordinator, rounded with the IPGS team every weekday; the Ward Team Coordinator entered orders including those for discharge and coordinated elements of discharge planning including home health services and durable medical equipment. Resident physicians and medical students rotated onto IPGS 1 month at a time. IPGS patients were mostly housed in a single pediatric acute care medicine floor alongside general pediatric hospitalist service patients, except in cases of limited ward capacity.

### Measures

Process measures included

1. discharge order time: the time of day when a patient’s discharge order was entered into the EMR;
2. physical discharge time: the time of day when a patient physically departed from the floor;
3. discharge response time: the elapsed time difference between discharge order time and physical discharge time.

The outcome measures were

1. percent of IPGS patients discharged before 1 pm with a goal of a 20% increase from preintervention baseline;
2. LOS index: the ratio of actual LOS to expected LOS.^10,11^ Actual LOS was a measured value which represented the elapsed time a patient was hospitalized. Expected LOS was a calculated value obtained from the hospital’s EMR based on national averages for diagnosis-related groups. The target value for LOS index was < 1, indicating an actual LOS shorter in duration than the expected LOS. The authors expected that if this study’s interventions were successful at improving discharge timeliness, LOS index would decrease. Our goal was a 20% decrease from preintervention baseline.

The balancing measure was

1. readmission rates within 30 days of discharge, commonly used in studies focused on the discharge process.

We collected these process, outcome, and balancing measures for all IPGS patients discharged during 3 periods:

1. The baseline “preintervention” period, from January 1 to June 1, 2016, lasting 153 days. Data were collected retrospectively.
2. The “postintervention” period from June 20 to December 31, 2016, lasting 195 days. Data were collected prospectively.
3. The “implementation” period from June 2 to June 19, 2016, during which time we introduced the interventions. Data were collected prospectively.

### Analysis

We obtained data from the Huron Healthcare ONTRAC dashboard (Huron Consulting Group, Chicago, IL), which unifies discharge, diagnosis, and LOS information from the EMR. This study used Lean and Six Sigma quality improvement methodologies. Control charts were generated using JMP statistical analysis software version 12.0 (SAS Institute, Cary, NC). Signals of special cause on control charts were identified using the Nelson rules.

### Preintervention Planning

**Fig. 1.**
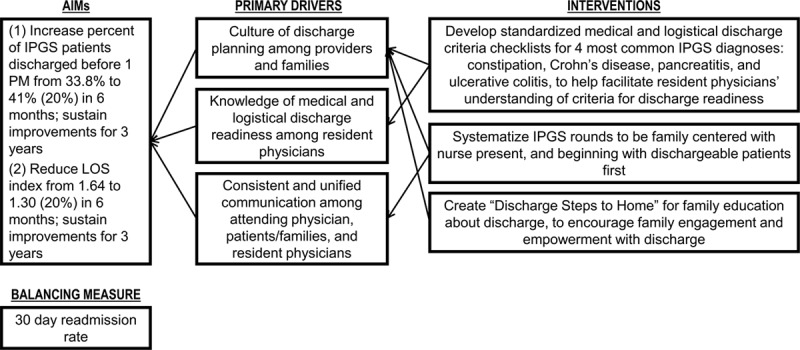
Key drivers diagram demonstrating information obtained through feedback from process stakeholders. Arrows demonstrate Interventions targeted to specific Key Drivers, and Key Drivers subsequently affecting aims.

The authors of this study met with members of the IPGS team to identify the current state by learning more about the service’s discharge process. These meetings included *Gemba* walks, a term from the original Japanese word *gembutsu* describing the in-person observation of work or the “real thing.”^12^ Gemba walks and interviews of staff allowed for greater understanding of IPGS operations, hospital workflow, team dynamics during rounds, the discharge process, and barriers to discharge. We organized information obtained from Gemba walks and interviews into a key drivers diagram that included our aims, primary drivers of discharge delays, and interventions (Fig. 1). We utilized a key drivers diagram template developed by the Institute for Healthcare Improvement (Boston, MA). We obtained buy-in and engagement at multiple levels, including nursing administration, medical unit leadership, hospital Six Sigma Black Belt champions, IPGS rounding team members, and the pediatric gastroenterology division chief.

### Interventions

Based on IPGS rounding team feedback, evidence from previous studies,^5,6,13^ experience with past interventions from the general pediatric hospitalist service, and key drivers, 3 interventions were designed and implemented to improve discharge timeliness.

First, rounds were systematized to occur in a family-centered manner in the patient’s room. The pediatric hospitalist teams at our institution had adopted family-centered rounds several years before our study, but IPGS had not adopted the family-centered rounding model. The IPGS rounding team resident physicians reported that when attending physicians and family or nursing communications occurred outside of rounds, communication gaps occurred. These communication failures led to confusion of the treatment and discharge plans. Rounds were modified to begin with dischargeable patients first so that discharge orders could be placed earlier in the day, and thus discharge activities could begin earlier as well. Finally, IPGS rounding team members requested nursing presence during rounds, particularly on the day of discharge, as they played a major role in coordinating discharge processes.

Second, families were educated about the role they played in discharge timeliness and encouraged to be actively involved. IPGS rounding team members reported that when patients and families were notified of the team’s intent to discharge them on that day, they sometimes requested to stay longer to eat lunch in the hospital or to wait for transportation. We placed a “Discharge Steps to Home” placard in each room to inform families of common day-of-discharge barriers, such as planning for the ride home, or the need for a school excuse note. We encouraged families to be proactive about addressing these discharge barriers.

Third, during *Gemba* walks, IPGS rounding team resident physicians reported not feeling confident in knowing when patients were ready for discharge. Therefore, standardized discharge criteria checklists were integrated into the Epic (Epic Systems, Verona, WI) EMR workflow to prioritize milestones toward discharge, and reduce ambiguity among IPGS rounding team members about discharge readiness. We implemented standardized discharge criteria checklists for the 4 most common IPGS discharge diagnoses from the preintervention baseline period. These diagnoses were Crohn’s disease (including perianal abscess/fistula, and obstruction), pancreatitis, ulcerative colitis, and constipation.

We based the medical discharge criteria for ulcerative colitis, Crohn’s perianal abscess/fistula, and Crohn’s obstruction on work from Wahbeh et al.^14,15^ For example, the medical discharge criteria for ulcerative colitis consisted of Pediatric Ulcerative Colitis Activity Index score < 35, afebrile for 24 hours, normal vital signs, sufficient oral intake and hydration, no intravenous medications, pain control with oral medications, and stable hemoglobin without need for transfusion in the previous 2 days. Medical discharge criteria for constipation were based on work from White et al.^4^ Pancreatitis discharge criteria, as well as the finalized criteria for all aforementioned diagnoses, were determined by our institution’s IPGS attending physician consensus in a manner similar to that of White et al.^4^ Logistical discharge criteria such as availability of transportation and acquiring new home prescriptions were included based on IPGS rounding team member feedback, as well as an author’s experience piloting a similar system for the adult general medicine inpatient service.

## RESULTS

**Fig. 2.**
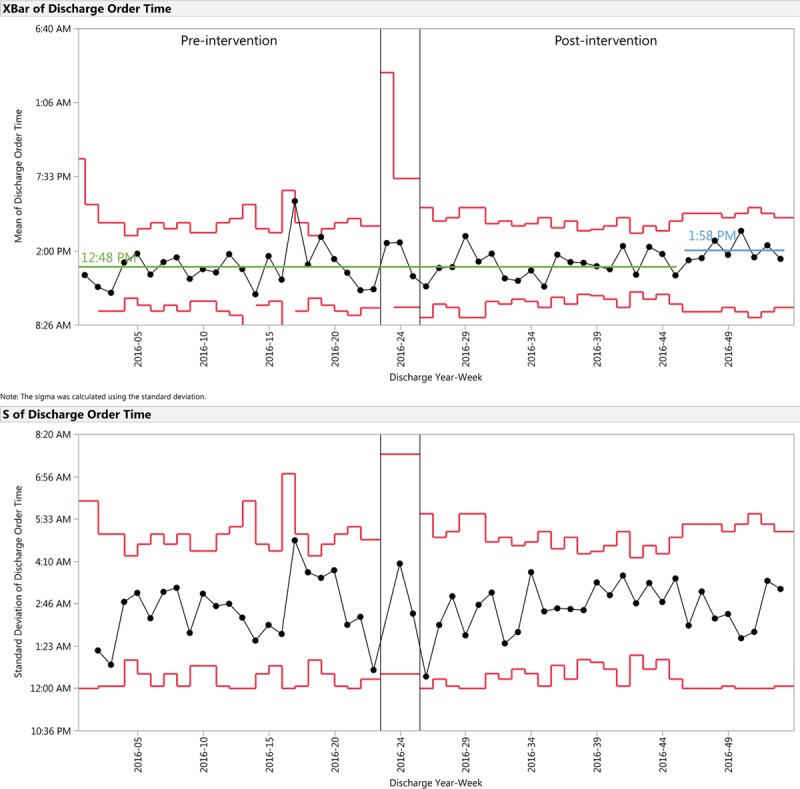
X-bar and S control chart demonstrating discharge order time for the preintervention and postintervention periods. Discharge order time is the time of day when the discharge order was placed in EMR. Preintervention mean centerline is indicated in green. Postintervention shifted centerline is indicated in blue.

**Fig. 3.**
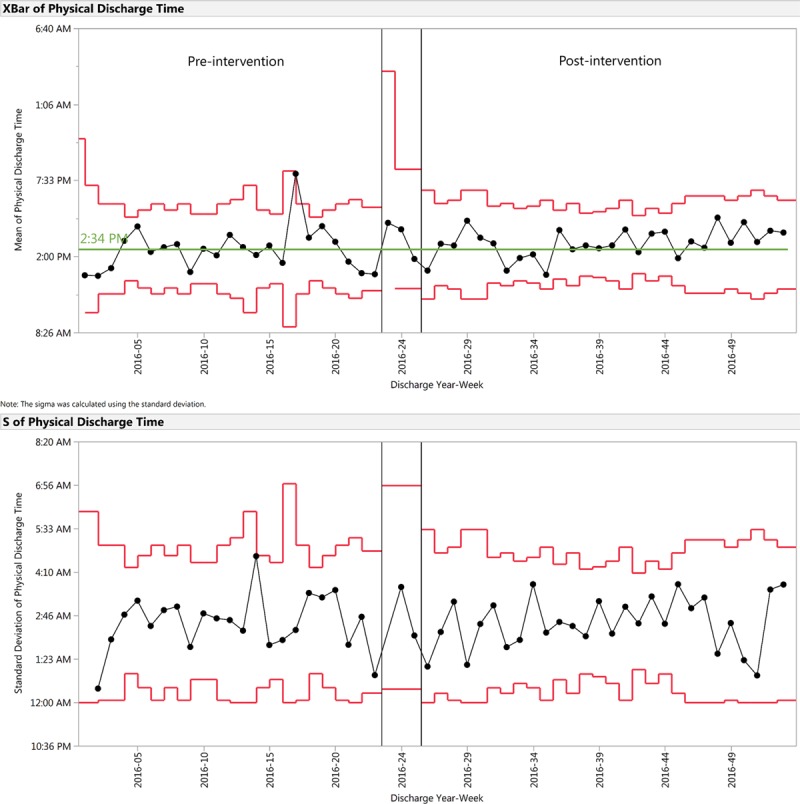
X-bar and S control chart demonstrating physical discharge time for the preintervention and postintervention periods. Physical discharge time is the time of day when a patient physically departed from the floor. Preintervention mean centerline is indicated in green. No postintervention shift was noted.

**Fig. 4.**
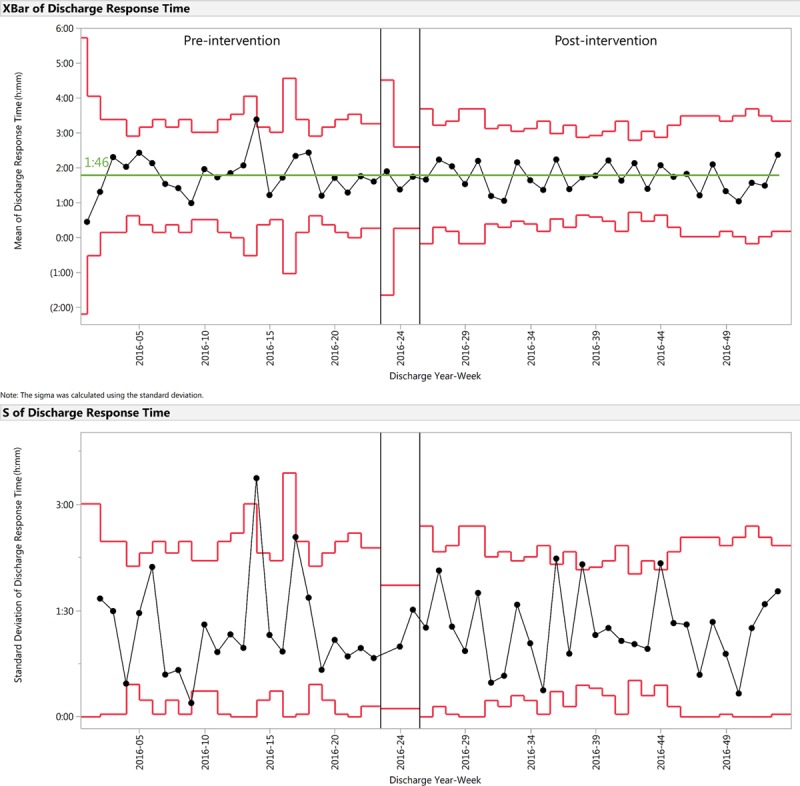
X-bar and S control chart demonstrating discharge response time for the preintervention and postintervention periods. Discharge response time is the elapsed time difference between discharge order time and physical discharge time. Preintervention mean centerline is indicated in green. No postintervention shift was noted.

In the preintervention period, there were 154 IPGS discharges (1.01 discharges per day), compared with 201 discharges (1.03 discharges per day) in the postintervention period. X-bar and S control charts during preintervention and postintervention periods for the process measures of discharge order time, physical discharge time, and discharge response time are shown in Figures 2, 3, and 4, respectively. Discharge order time shifted from 12:48 pm pre-intervention, to 1:58 pm during weeks 46–53 of 2016.

**Fig. 5.**
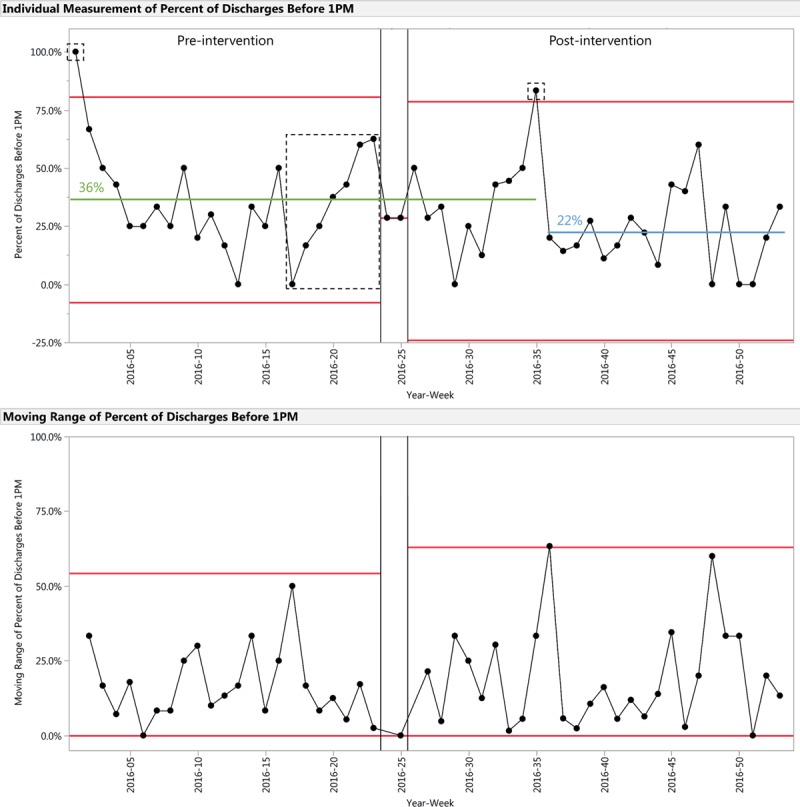
Shewhart individuals control chart demonstrating percent of discharges before 1 pm for the preintervention and postintervention periods. Preintervention mean centerline is indicated in green. Postintervention shifted centerline is indicated in blue. Signals of special cause are identified in the dotted rectangles.

For the outcome measure of the percent of discharges before 1 pm, a Shewhart individuals control chart is shown in Figure 5. Percent of discharges before 1 pm shifted from 36% pre-intervention to 22% during weeks 36–53 of 2016, a 39% decrease. We identified multiple signals of special cause:

1. Week 1 of 2016
2. Weeks 17–23 of 2016
3. Week 35 of 2016

**Fig. 6.**
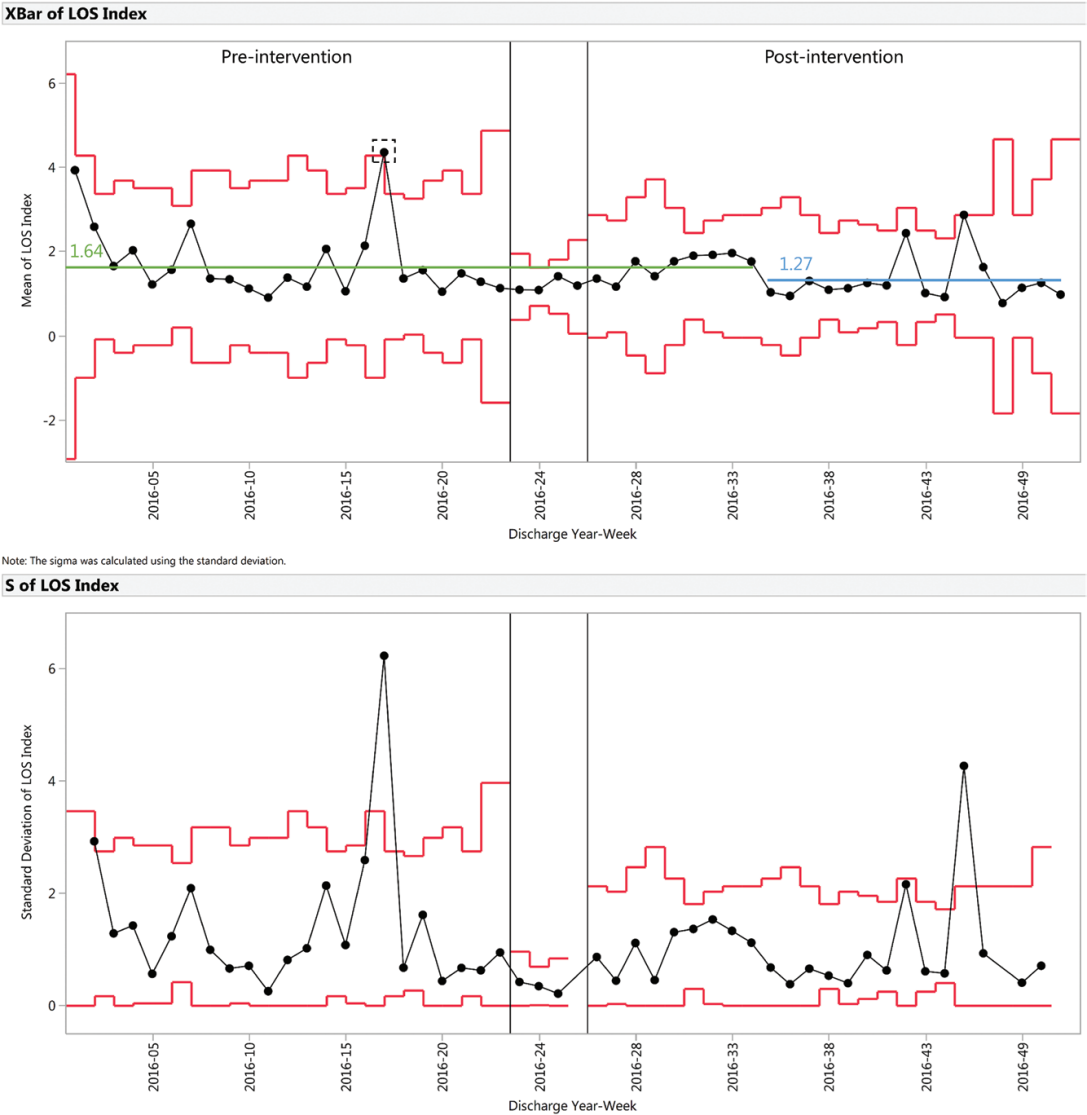
X-bar and S chart demonstrating LOS index for the preintervention and postintervention periods. LOS index is calculated as the ratio of actual LOS to expected LOS. Preintervention mean centerline is indicated in green. Postintervention shifted centerline is indicated in blue. A signal of special cause is identified in the dotted rectangle.

For the outcome measure of LOS index, an X-bar and S control chart is shown in Figure 6. LOS index shifted from 1.64 pre-intervention to 1.27 during weeks 35–51 of 2016, a 23% decrease. We identified 1 signal of special cause:

1. Week 17 of 2016

The balancing measure of 30-day readmission rate was 15.3% in the preintervention period and 18.1% in the postintervention period (*P* = 0.54).

## DISCUSSION AND CONCLUSIONS

With this study, we designed, implemented, and studied interventions to improve the timeliness of IPGS discharges. To our knowledge, this is the first study that has focused on improving discharge timeliness on a pediatric inpatient subspecialty service, whereas most other work has focused on general hospitalist or inpatient services.

We did not find significant improvements in the 3 process measures. There was not a significant improvement in outcome measure of discharges before 1 pm. However, the outcome measure of LOS index did improve, indicating that patients’ actual LOS declined relative to expected LOS. The decline in LOS index speaks to an overall postintervention improvement of the IPGS discharge process. We also postulate that the LOS index improvement reflected increased willingness to engage in the discharge process throughout hospitalization, a major cultural change, although this was not directly studied. Another explanation for the lack of improvement in the process measures and percent of discharges before 1 pm is that, although discharge order times and physical discharge times did not occur earlier between the preintervention and postintervention periods, discharges occurred nearly 1 day sooner in patients’ overall hospitalization. Therefore, even though patients were discharged later in the day, their aggregate length of hospitalization declined. We believe this is attributable to the development of a discharge-oriented culture in which discharge orders were placed soon after patients became medically and logistically ready, even if this meant orders were placed long after 1 pm; therefore, patients would still leave the hospital earlier than if their discharge orders were instead placed the next day before 1 pm.

As noted in the Results section, there were 3 signals of special cause identified in percent of discharges before 1 pm (Fig. 5). The signal of special cause in week 1 of 2016 is attributable to there being only one discharge that week, which occurred at 12:37 pm; similarly in week 35 of 2016 (post-intervention), there were 6 total discharges, of which only one occurred after 1 pm. We attribute the signal of special cause at weeks 17–23 of 2016 (pre-intervention) to the Hawthorne effect; as described in the Methods section, during preintervention planning, IPGS attending physicians came to be aware of this study around weeks 17–23 of 2016, learned of its aims, and were consulted to finalize medical discharge criteria for the most common IPGS diagnoses. IPGS attending physicians may have modified their behaviors in response to the awareness of the fact that their rounding and discharge practices were to be observed, leading to an increase in the percent of discharges before 1 pm.

With patient discharge orders being placed later in the day, physical discharge time delays would have been exacerbated by decreased hospital staffing toward the end of the day, for example, nurses, patient transporters. As a result of delayed physical discharge time, discharge response time would subsequently worsen too. In the presence of these delays, we found that patients’ total hospitalization length was shortened following the implementation of our interventions, as indicated by the downward shift in LOS index (Fig. 6). We postulate a few factors that may have contributed to the 10-week delay between the start of the intervention and the occurrence of the downward shift: incomplete or slow adoption of the interventions by IPGS rounding team members; lack of familiarity with the newly developed medical and logistical discharge criteria; and lack of knowledge on how to use discharge criteria properly in the EMR workflow. We attribute the signal of special cause in week 17 of 2016 (pre-intervention) to a single patient’s outlying discharge with a LOS index of 11.5, out of 3 total discharges that week.

As a measure of medically unnecessary discharge delay, investigators have used the reduction in LOS index as a proxy measure of unnecessary cost and waste. Framed in terms of days, the mean actual LOS declined from 5.7 days to 4.7 days (*P* = 0.055). Reducing actual LOS by 1 day for the postintervention population of 201 patients, given average hospitalization cost per inpatient day in North Carolina of $1,854,^16^ can yield savings of approximately $373,000.

Overall, this study yielded mixed results. We did not accomplish our first aim to increase discharges before 1 pm. However, our second aim to improve the LOS index was met. The process measure of discharge order time worsened, whereas physical discharge time and discharge response time remained unchanged. We believe this is due to the mixed effectiveness of our interventions. In terms of causal pathways linking interventions to measures, we expected that systematizing rounds would improve discharge order time as beginning rounds with dischargeable patients first would allow for their discharge orders to be placed earlier in the day. This intervention likely did not improve the timeliness of discharge order entry as the IPGS rounding team may have deferred orders until after rounds as a result of cultural, communication, or other barriers not identified or addressed in this study. Additionally, we expected the streamlined communication among IPGS rounding team members, and with families, during family-centered rounds would help make discharge planning transparent such that day-of-discharge barriers could be minimized, thus improving physical discharge time.

In contrast, we believe family education and empowerment about discharge and standardized discharge criteria checklists were likely more effective interventions as they both contributed to a discharge-oriented culture among patients, parents, and providers. This change possibly led to an improvement in LOS index.

Experts promote the idea that hospitals should aim to discharge patients as close as possible to when they are medically ready for discharge.^4^ In addition to increasing hospital throughput and capacity, timely discharges can incrementally increase hospital revenue while reducing costs. Hospitalized patients are typically billed by midnights stayed, for which the hospital receives a fixed payment. When the discharge occurs sooner since the elapsing of a new midnight threshold, costs of additional meals, nursing personnel, and ancillary services are reduced, thus increasing revenue available to the hospital. Also, when patient beds become available and new patients are admitted earlier in the day, potentially more experienced staff and personnel are available, and so tests and procedures can commence instead of waiting until the next hospital day.

Key lessons from this study included the importance of garnering early stakeholder feedback and buy-in at all levels. Understanding how processes work on an academic inpatient service through *Gemba* walks and modifying interventions based on real-time feedback were particularly effective.

There are several limitations to this work. This study took place at a single hospital on a single inpatient specialty service. This fact may limit the generalizability of the results. Another limitation is that IPGS attending physicians’ knowledge of and participation in the development of interventions during preintervention planning may have resulted in the Hawthorne effect influencing our results. As discussed previously, we believe the 3 interventions to have been of varying effectiveness. Because all interventions were purposely implemented nearly simultaneously due to time constraints, we are unable to determine the effect on process and outcome measures that each one had individually. Finally, changes in process, outcome, and balancing measures may be unrelated to the interventions. However, we do not believe this is likely as there was a temporal association between changes and implementation. Furthermore, although LOS index did improve among IPGS patients, it remained constant among non-IPGS patients housed on the same primary pediatric acute medicine floor, at 1.88, both pre-intervention and post-intervention (*P* = 0.99). Additionally, there were no other major changes or initiatives on IPGS, which we identified, such as with patient population or healthcare personnel.

## DISCLOSURE

The authors have no financial interest to declare in relation to the content of this article.
